# The role of cardiac magnetic resonance imaging in the assessment of systemic lupus erythematosis (SLE)

**DOI:** 10.1186/1532-429X-11-S1-P199

**Published:** 2009-01-28

**Authors:** Wendy Norman, Sean G O'Neill, Simon Woldman, Frederique Bailliard, Anisur Rahman, Andrew M Taylor

**Affiliations:** 1 UCL Institute of Child Health, London, UK; 2 Centre for Rheumatology Research, UCL, London, UK; 3 The Heart Hospital, UCLH, London, UK

**Keywords:** Systemic Lupus Erythematosis, Cardiac Magnetic Resonance, Myocarditis, Late Gadolinium Enhancement, Cardiac Magnetic Resonance Imaging

## Objectives

To define the cardiac magnetic resonance (MR) appearances of cardiac disease in patients with SLE and known coronary or cerebrovascular disease, and compare theses findings with those seen on trans-thoracic echocardiography (TTE).

## Background

Cardiovascular disease (CVD) is a major cause of morbidity and mortality in patients with SLE. It is increasingly recognised that accelerated atherosclerosis contributes to increased risk of myocardial ischaemia and/or stroke in this population. Autopsy and echo studies have documented numerous abnormalities in patients with SLE; however it remains unclear whether these abnormalities are an important contributor to mortality and morbidity, and whether they are related to early development of coronary artery disease.

## Methods

Cardiac MR assessment including late gadolinium enhancement (LGE) was performed on 22 patients meeting 4 or more of the revised ACR criteria for SLE. Imaging performed at 1.5 T (Avanto, Siemens, Erlangen, Germany). 11 patients had previous CVD and were matched with 11 control patients with no history of CVD. Control patients were matched for age, gender, race and SLE disease duration. TTE was performed at the same visit. The Research Ethics Committee approved the study and all participants gave informed consent to take part.

## Results

**Figure 1:**
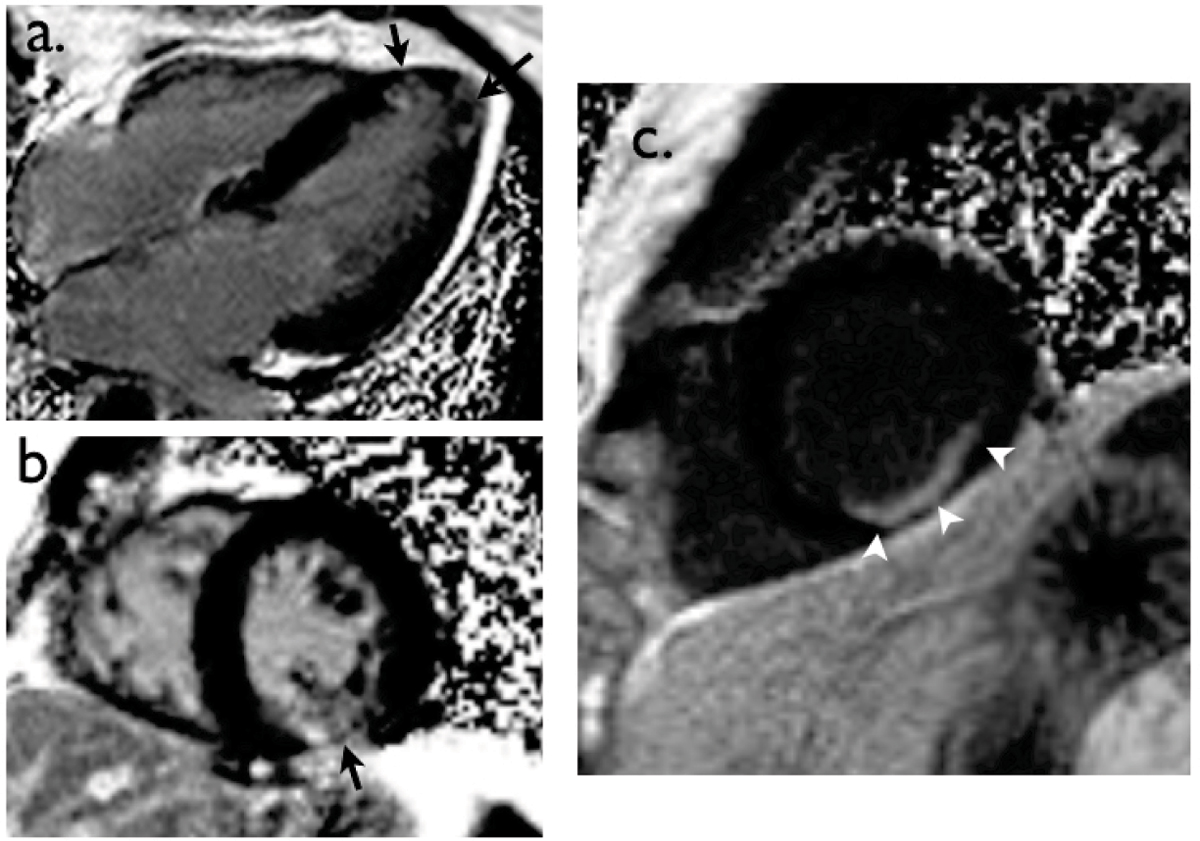
**Unexpected areas of LGE:** a&b, patchy LGE in a non-coronary artery distribution pattern *(arrows)*, consistent with possible myocarditis – a, 4-chamber view showing sptal and apical LGE and b, short-axis view showing inferior LGE; c, subendocardial LGE in a circumflex coronary artery distribution *(arrowheads)*, consistent with silent AMI in a patient with APS.

Of the subjects in the known CVD group, 5 had areas of LGE on the MR images: 4 LGE patterns were consistent with myocardial infarction, whilst in 1 patient (history of stroke) there was patchy LGE in a non-coronary distribution (Figure 1a &1b).

One subject, with known antiphospholipid antibody syndrome (APS) but no history of CVD, had LGE suggestive of a previous infarct (Figure 1c). This was not evident on TTE. One patient purported to have had a myocardial infarction had no LGE. TTE myocardial abnormalities were detected on 4 of the 6 subjects that had LGE.

## Conclusion

In this small study, we demonstrate that cardiac MR can detect changes in the myocardium of patients with SLE that are not evident on TTE. The 2 imaging modalities were carried out on the same day to avoid the possibility of new abnormalities developing between the dates of the MR and TTE. Three of the 22 patients we studied (14%) had diagnostic MR scans that could potentially influence their long-term management and prognosis. Two patients with no clinical history of cardiac disease had areas of unexpected myocardial fibrosis, one with a pattern suggestive of coronary artery disease, and one with multiple areas of myocardial scarring, perhaps suggestive of myocarditis. Both these patients had normal TTE. Conversely, 1 patient with a supposed acute myocardial infarction had no evidence of myocardial fibrosis on MR scanning, placing this patient in a good prognostic group for the future. Further studies of cardiac MR in patients with SLE are warranted to investigate these preliminary findings.

